# Longitudinal Trends and Socioeconomic Disparities in Testicular Torsion and Orchiectomy Spanning the COVID-19 Era: A Pediatric Health Information System Database Study

**DOI:** 10.7759/cureus.106539

**Published:** 2026-04-06

**Authors:** Jordan Walsh, John H Makari, Claudia Berrondo

**Affiliations:** 1 Urology, University of Nebraska Medical Center, Omaha, USA; 2 Surgery/Pediatric Urology, University of Nebraska Medical Center, Omaha, USA; 3 Surgery/Pediatric Urology, Children's Nebraska, Omaha, USA; 4 Surgery/Pediatric Urology, Children’s Nebraska, Omaha, USA

**Keywords:** covid-19 pandemic, orchiectomy, pediatric, pediatric health information system, testicular torsion

## Abstract

Introduction: The impact of COVID-19 on testicular torsion frequency, management, and outcomes remains underexplored, with existing studies showing inconsistent results. This study aims to assess the frequency of testicular torsion, orchiectomy rates, and associated demographic and socioeconomic risk factors before, during, and after the pandemic.

Methods: The U.S.-based Pediatric Health Information System (PHIS) database was queried to identify patients (1-18 years) diagnosed with testicular torsion (International Classification of Diseases, 10th Revision (ICD-10) codes N44.0 and N44.01) who underwent surgical treatment (Current Procedural Terminology (CPT) codes 54520, 54550, 54560, 54640, 54650, 54692, 54600, and 54620) between March 13, 2018, and June 30, 2024. We analyzed demographic data, calculated frequencies of torsion and rates of orchiectomy, and compared findings across three periods: pre-COVID-19 (March 13, 2018, to March 13, 2020), during COVID-19 (March 14, 2020, to March 13, 2021), and post-COVID-19 (March 14, 2021, to June 30, 2024).

Results: Of the 12,399 patients with testicular torsion, 2,792 (22.5%) underwent orchiectomy. The number of testicular torsion cases per month increased before and during the pandemic, then plateaued post-pandemic. The number of cases per month was highest in those aged 12-14 years, followed by ≤12 years, and was higher in non-Hispanic White individuals.

Orchiectomy rates showed a slight downward trend over time. Rates were highest in younger patients (≤12 years), were consistently higher in non-White patients than in White patients, and were lower in patients with commercial insurance compared with other insurance types. Adjusted odds ratios for orchiectomy were higher in younger patients, non-White patients, and patients with Medicaid insurance across all time periods.

Discussion: The observed monthly frequency of testicular torsion trended upward before and during the COVID-19 pandemic and subsequently stabilized in the post-pandemic period, while orchiectomy rates declined slightly and were not affected by the pandemic. Younger and non-White patients had higher odds of orchiectomy, whereas patients with commercial insurance had lower odds. Limitations include those inherent to administrative databases, lack of true incidence data, and potential regional differences in healthcare access, which may affect generalizability.

Conclusion: The monthly frequency of testicular torsion increased before and during the COVID-19 pandemic and subsequently plateaued in the post-pandemic period. The COVID-19 pandemic did not significantly alter orchiectomy rates. Younger age, non-White race, and non-commercial insurance were associated with increased odds of orchiectomy, and this effect was not altered by the pandemic.

## Introduction

Testicular torsion is a urologic emergency in which the spermatic cord twists, leading to ischemia of the testicle. There is a bimodal distribution of testicular torsion, with a peak in the first year of life (neonatal testicular torsion) and a second, larger peak in adolescents aged 12-18 years [1]. A 2005 study estimated its incidence at 4.5 cases per 10,000 males per year in the United States [2]. Testicular torsion is a clinical diagnosis, with hallmark symptoms including acute testicular pain, scrotal swelling, scrotal erythema, testicular tenderness, nausea, vomiting, and abnormal testicular positioning [3]. Testicular torsion may also present atypically, lacking hallmark symptoms and instead manifesting with subtle findings such as lower abdominal or groin pain. In rare cases, bilateral torsion can occur, often contributing to delayed diagnosis and reduced rates of testicular salvage [4].

Rapid assessment and treatment are critical for testicular salvage; every 10-minute delay in the Emergency Department increases the risk of a nonviable testis by 4.8% [5]. Time from presentation to detorsion is an independent predictor of testicular survival, and the salvage rate is highest within 4-8 hours of symptom onset [2]. Management within 6 hours preserves 90%-100% of testes, compared with 20%-50% at 6-12 hours and 1%-10% at 12-24 hours [6]. Surgical intervention involves detorsion with orchiopexy or orchiectomy if the testis is nonviable, along with contralateral orchiopexy to reduce the risk of asynchronous torsion. National studies report orchiectomy rates in pediatric patients ranging from 32% to 42% [2,7,8].

SARS-CoV-2 was first identified in 2019 and declared a global pandemic, COVID-19, by the World Health Organization (WHO) on March 11, 2020 [9]. In response, a nationwide state of emergency was declared by the United States president on March 13, 2020. To limit the spread of SARS-CoV-2, the Centers for Disease Control and Prevention (CDC) implemented social distancing guidelines and stay-at-home orders, resulting in widespread closures of schools and workplaces. These restrictions contributed to increased avoidance of medical facilities, which has been linked to higher morbidity and mortality for acute and chronic conditions [10,11]. Current data remain inconclusive regarding the impact of the COVID-19 pandemic on the presentation and outcomes of pediatric patients with testicular torsion [3,9,12-20].

Our objectives were to investigate temporal trends in the monthly frequency of non-neonatal testicular torsion and orchiectomy rates in pediatric patients before, during, and after the COVID-19 pandemic using the Pediatric Health Information System (PHIS) database. Additionally, we sought to identify demographic and socioeconomic factors associated with disparities in orchiectomy rates in this cohort during the same timeframe. Although most COVID-19 precautions are no longer in place and the virus has become part of daily life, there remains important historical and epidemiological value in examining its impact on testicular torsion. Such insights not only enhance our understanding of how a global pandemic can influence emergent urologic conditions but may also help better prepare healthcare providers for future public health crises.

## Materials and methods

Data source and acquisition

The Pediatric Health Information System (PHIS) is a national administrative database composed of clinical and resource utilization data for encounters at 49 non-profit, tertiary pediatric hospitals in the United States. All data are deidentified and undergo reliability and validity checks by the Children’s Hospital Association (Lenexa, Kansas). Permission to report deidentified data from the PHIS database was obtained from the Children’s Hospital Association, and this study was exempt from Institutional Review Board approval.

The PHIS database was queried for records of pediatric patients with testicular torsion, defined as having both International Classification of Diseases, 10th Revision (ICD-10) diagnosis codes for testicular torsion (N44.0 and N44.01) and Current Procedural Terminology (CPT) codes associated with surgery for treatment of acute testicular torsion (54520, 54550, 54560, 54640, 54650, 54692, 54600, and 54620), between March 13, 2018, and June 30, 2024. Patients <1 year of age were not included in the query in order to focus exclusively on non-neonatal testicular torsion. Deidentified data were extracted from the PHIS database. To ensure data integrity, the cohort was restricted to hospitals maintaining continuous reporting throughout the study period. The following variables were collected: age at the time of surgery (prepubertal (≤12 years), pubertal (12-14 years), late pubertal (14-15 years), and postpubertal (≥15 years)), race (White, non-White), ethnicity (Hispanic/Latino, not Hispanic/Latino), insurance type (Medicaid, Commercial, Self/other), and hospital location (West, Midwest, South, and Northeast). The non-White category was not further subdivided due to the small sample sizes of individual racial subgroups. Therefore, they were combined to allow for more meaningful analysis. The self/other insurance category encompassed all remaining insurance types, including unspecified and government-issued plans.

Time periods were divided into pre-COVID-19 (March 13, 2018, to March 13, 2020), COVID-19 (March 14, 2020, to March 13, 2021), and post-COVID-19 (March 14, 2021, to June 30, 2024). The pre-COVID-19 period was selected as a baseline for comparison with the pandemic period. March 14, 2020, was chosen as the start of the COVID-19 period following the nationwide state of emergency declared on March 13, 2020. Although COVID-19 remains present, March 13, 2021, was designated as the start of the post-COVID-19 period, reflecting a new phase in the pandemic in the United States with widespread vaccine availability, changes in public health measures, social behavior, and healthcare access.

Outcomes and variables

Primary outcomes were the number of cases of testicular torsion per month, and orchiectomy rates. Potential predictive factors associated with orchiectomy included age, race, ethnicity, insurance type, time period of surgery, and hospital location.

Statistical analysis

Baseline demographic and clinical characteristics were summarized using descriptive statistics. Locally estimated scatterplot smoothing (LOESS) curves were drawn to evaluate the daily percentage of orchiectomy cases among total testicular torsion cases, to show differences across factor levels. Main effects for five fixed factors (age, race, ethnicity, insurance, and region) and their interactions with time (before, during, and after the COVID-19 pandemic) were evaluated with a binary response in a generalized linear mixed model (GLMM) with a logit link. A code representing the regional hospitals was applied as a random effect to account for multiple observations within each hospital. Significance for fixed effects was determined using Type III tests, with p-values calculated via F-tests. Comparisons across time segments were made using least squares means (LS-means) to provide predicted probabilities and 95% confidence intervals, adjusted for the random effect of location. Odds ratios comparing the proportions of orchiectomy across the three time periods and across levels of the factors were evaluated. All analyses were performed using SAS System for Windows, version 9.4 (© 2016, Cary, North Carolina). A p-value <0.05 was considered statistically significant.

## Results

Patient characteristics

Over the study period, we identified a total of 12,399 patients with testicular torsion, and orchiectomy was performed in 2,792 (22.5%). Demographics are outlined in Table 1. The majority of patients were ≤12 years (3,773, 30.4%) or 12-14 years (4,589, 37%). The majority of patients identified as White (6,617, 53.4%) and non-Hispanic or Latino (8,986, 72.5%). Most patients had commercial insurance (5,307, 42.8%). The South was the most common hospital location (5,282, 42.6%).

**Table 1:** Demographics of patients with testicular torsion.

Variables	Time Period			
	Pre-COVID-19, n (%) (N = 2963)	COVID-19, n (%) (N = 1962)	Post-COVID-19, n (%) (N = 7474)	Total, n (%) (N = 12,399)
Age (Years)				
≤12	983 (33.2)	575 (29.3)	2215 (29.6)	3773 (30.4)
12-14	1054 (35.6)	769 (39.2)	2766 (37)	4589 (37)
14-15	429 (14.5)	275 (14)	1149 (15.4)	1853 (14.9)
>15	497 (16.8)	343 (17.5)	1344 (18)	2184 (17.6)
Ethnicity				
Hispanic or Latino	639 (21.6)	399 (20.3)	1747 (23.4)	2785 (22.5)
Not Hispanic or Latino	2178 (73.5)	1477 (75.3)	5331 (71.3)	8986 (72.5)
Missing	146 (4.9)	86 (4.4)	396 (5.3)	628 (5.1)
Race				
Non-White	832 (28.1)	564 (28.7)	2286 (30.6)	3682 (29.7)
White	1567 (52.9)	1057 (53.9)	3993 (53.4)	6617 (53.4)
Missing	564 (19)	341 (17.4)	1195 (16)	2100 (16.9)
Primary source of payment				
Commercial	1450 (48.9)	1040 (53)	3708 (49.6)	6198 (50)
Medicaid	1298 (43.8)	794 (40.5)	3215 (43)	5307 (42.8)
Self/Other	185 (6.2)	120 (6.1)	534 (7.1	839 (6.8)
Missing	30 (1)	8 (0.4)	17 (0.2)	55 (0.4)
Region				
Northeast	328 (11.1)	248 (12.6)	1105 (14.8)	1681 (13.6)
Midwest	783 (26.4)	514 (26.2)	1778 (23.8)	3075 24.8)
South	1302 (43.9)	833 (42.5)	3147 (42.1)	5282 (42.6)
West	550 (18.6)	367 (18.7)	1444 (19.3)	2361 (19)

Testicular torsion frequency by patient demographics

**Figure 1:**
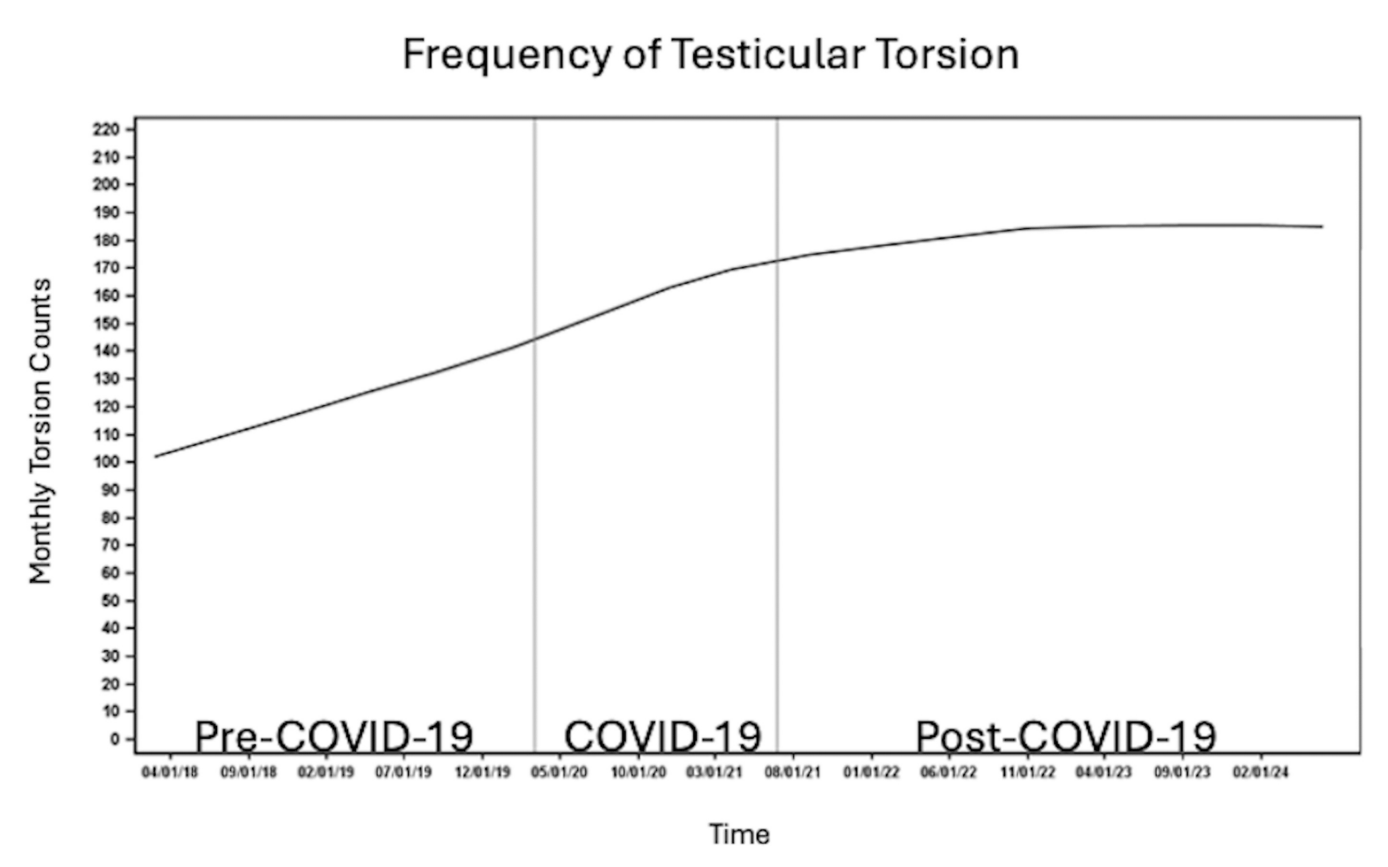
Number of cases per month of testicular torsion before, during and after the COVID-19 pandemic.

**Figure 2:**
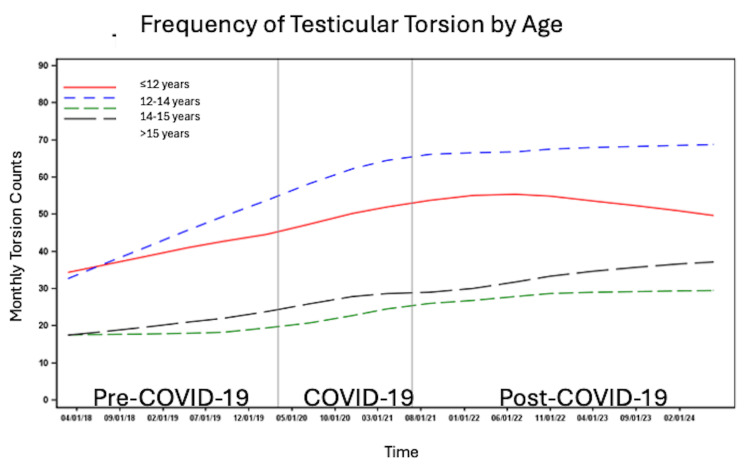
Number of cases per month of testicular torsion before, during and after the COVID-19 pandemic by patient age.

Trends in testicular torsion were examined using visible graphical trends only due to the lack of true incidence data for testicular torsion. The number of patients per month with testicular torsion steadily increased before and during the COVID-19 pandemic, followed by a plateau post-COVID-19 (Figure 1). The highest number of cases per month was observed in the 12-14 year age group across all time periods, followed by patients ≤12 years old, with the lowest number of patients per month in the 14-15 and >15 year age groups (Figure 2).

**Figure 3:**
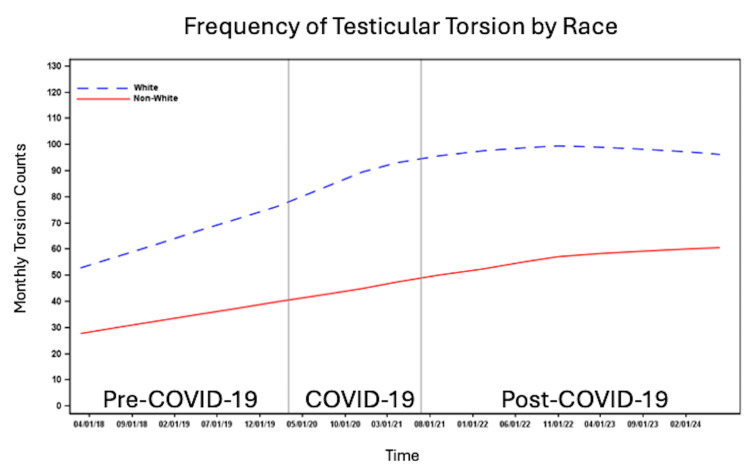
Number of cases per month of testicular torsion before, during and after the COVID-19 pandemic by race.

**Figure 4:**
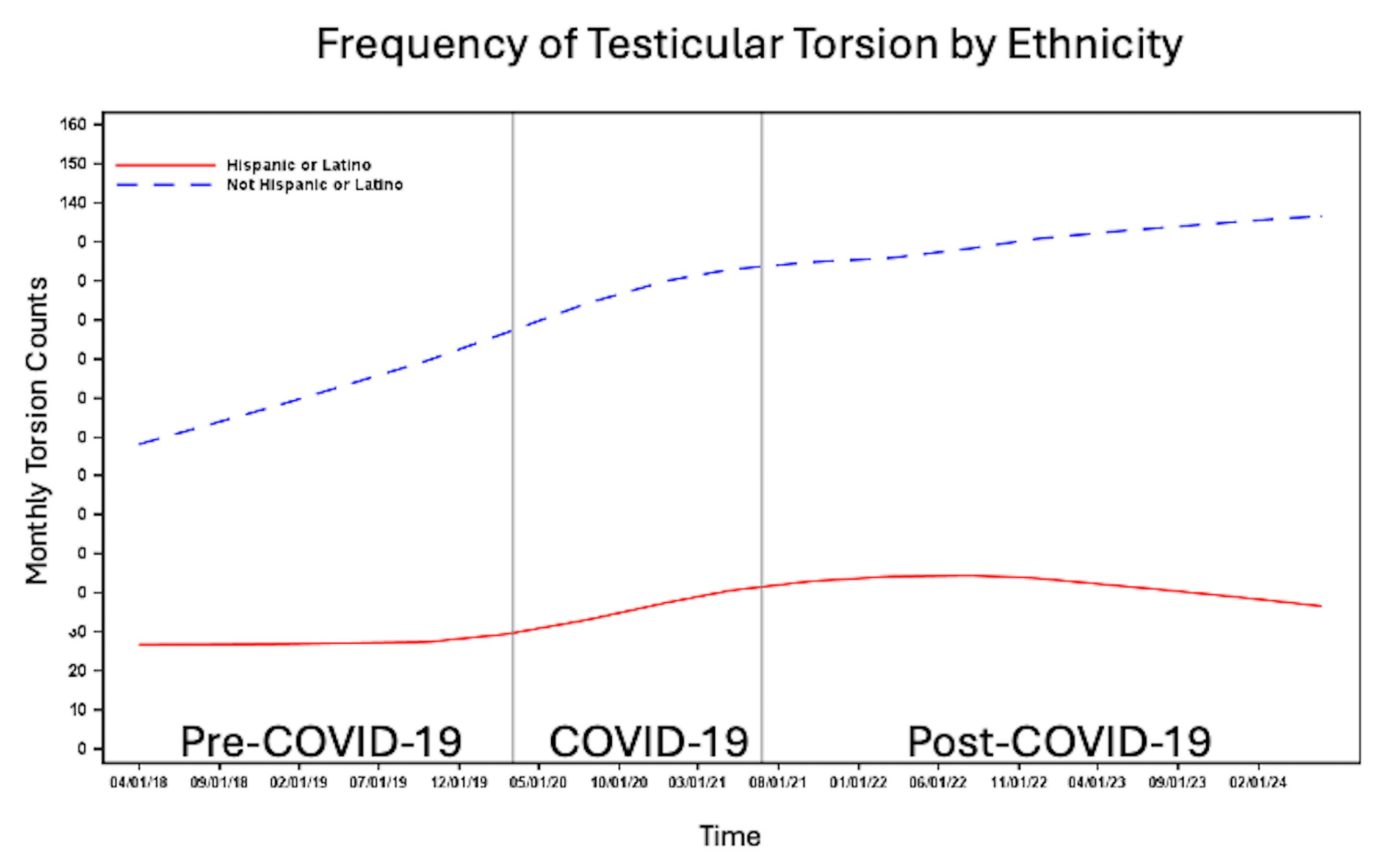
Number of cases per month of testicular torsion before, during and after the COVID-19 pandemic by ethnicity.

**Figure 5:**
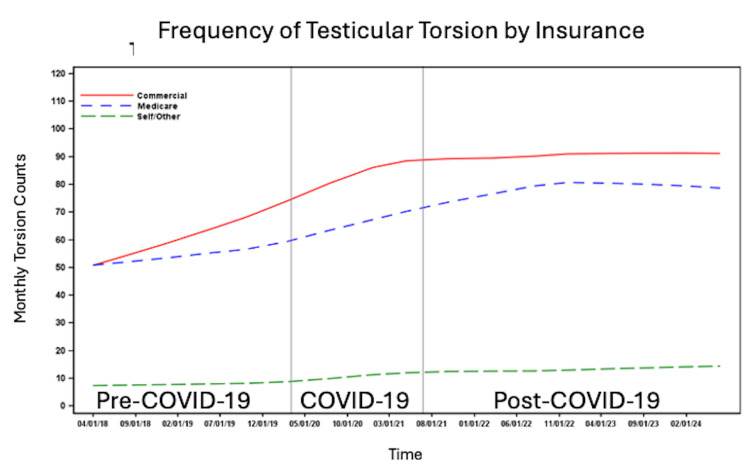
Number of cases per month of testicular torsion before, during and after the COVID-19 pandemic by insurance type.

**Figure 6:**
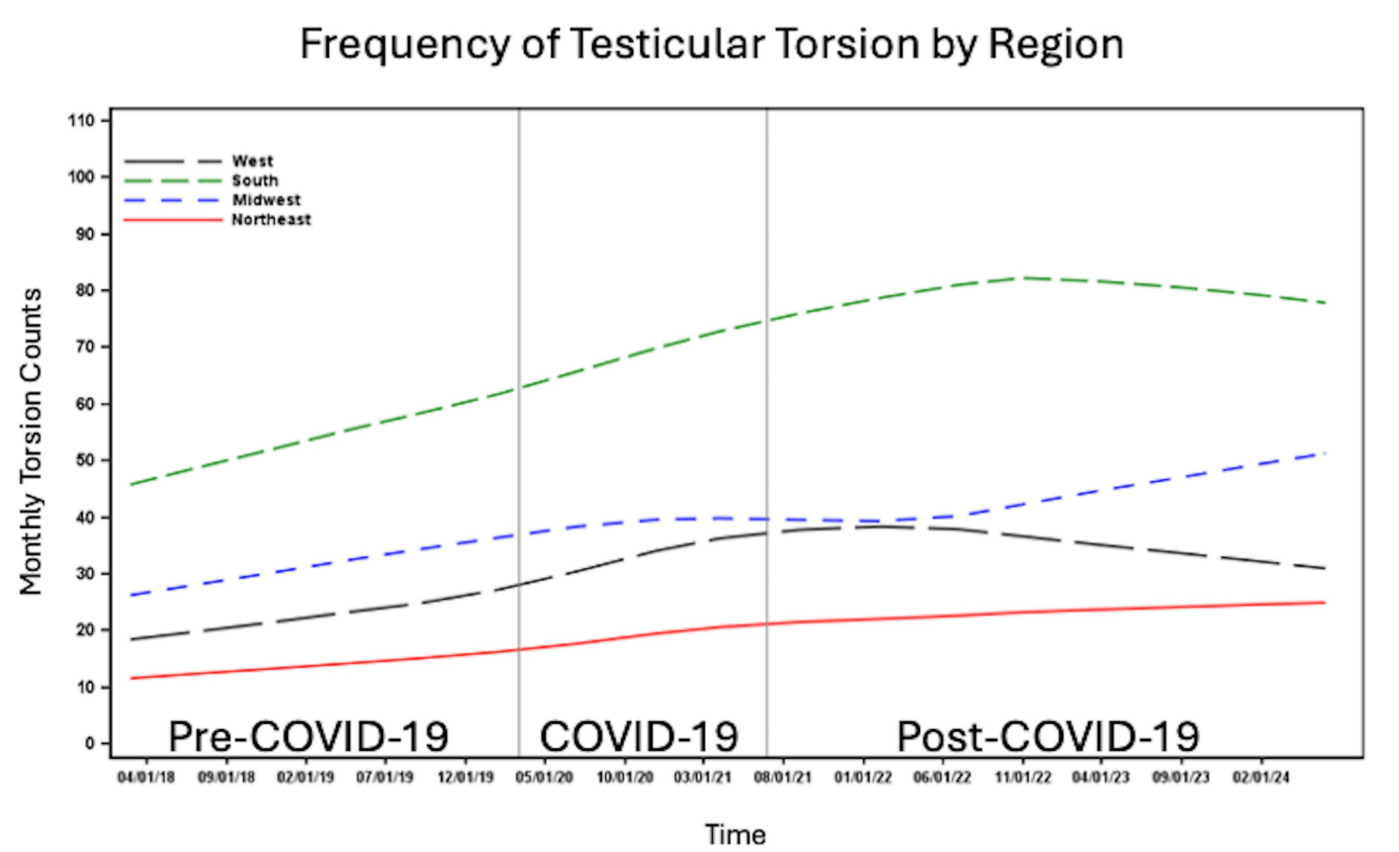
Number of cases per month of testicular torsion before, during and after the COVID-19 pandemic by geographic region.

The number of patients per month with testicular torsion was higher among White patients compared to non-White patients (Figure 3) and among non-Hispanic/Latino individuals compared to Hispanic/Latino individuals (Figure 4). The number of cases per month increased across all time periods for patients with commercial insurance, Medicaid, and self-pay/other insurance. However, the highest number of cases per month was observed among those with commercial insurance, followed by Medicaid, with the lowest number of cases per month in the self-pay/other insurance group (Figure 5). The number of cases per month was consistently highest in the South and consistently lowest in the Northeast across all time periods compared to the West and Midwest (Figure 6).

Orchiectomy rates by patient demographics

Age

**Figure 7:**
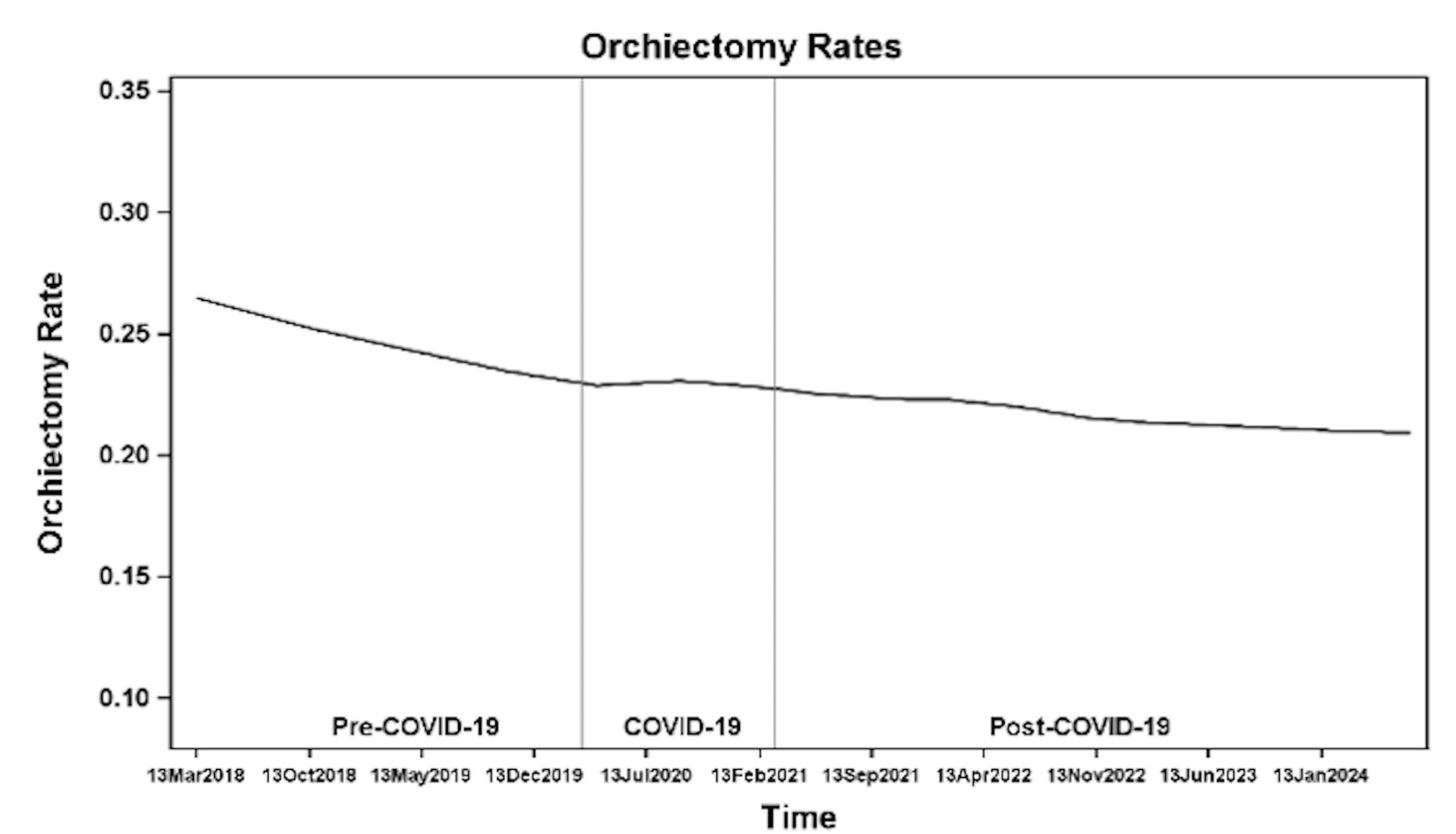
Orchiectomy rates before, during, and after the COVID-19 pandemic.

**Figure 8:**
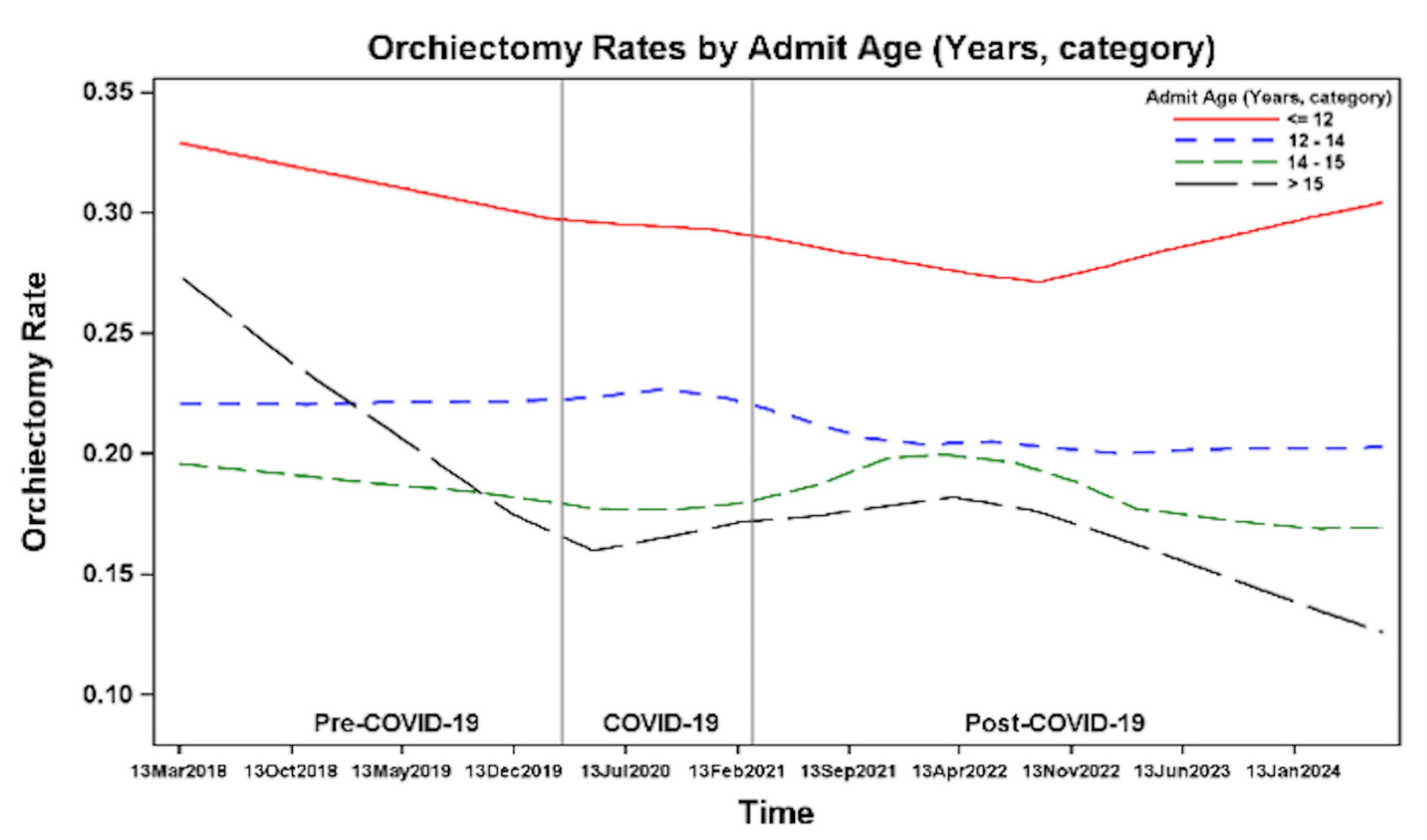
Orchiectomy rates before, during, and after the COVID-19 pandemic.

**Figure 9:**
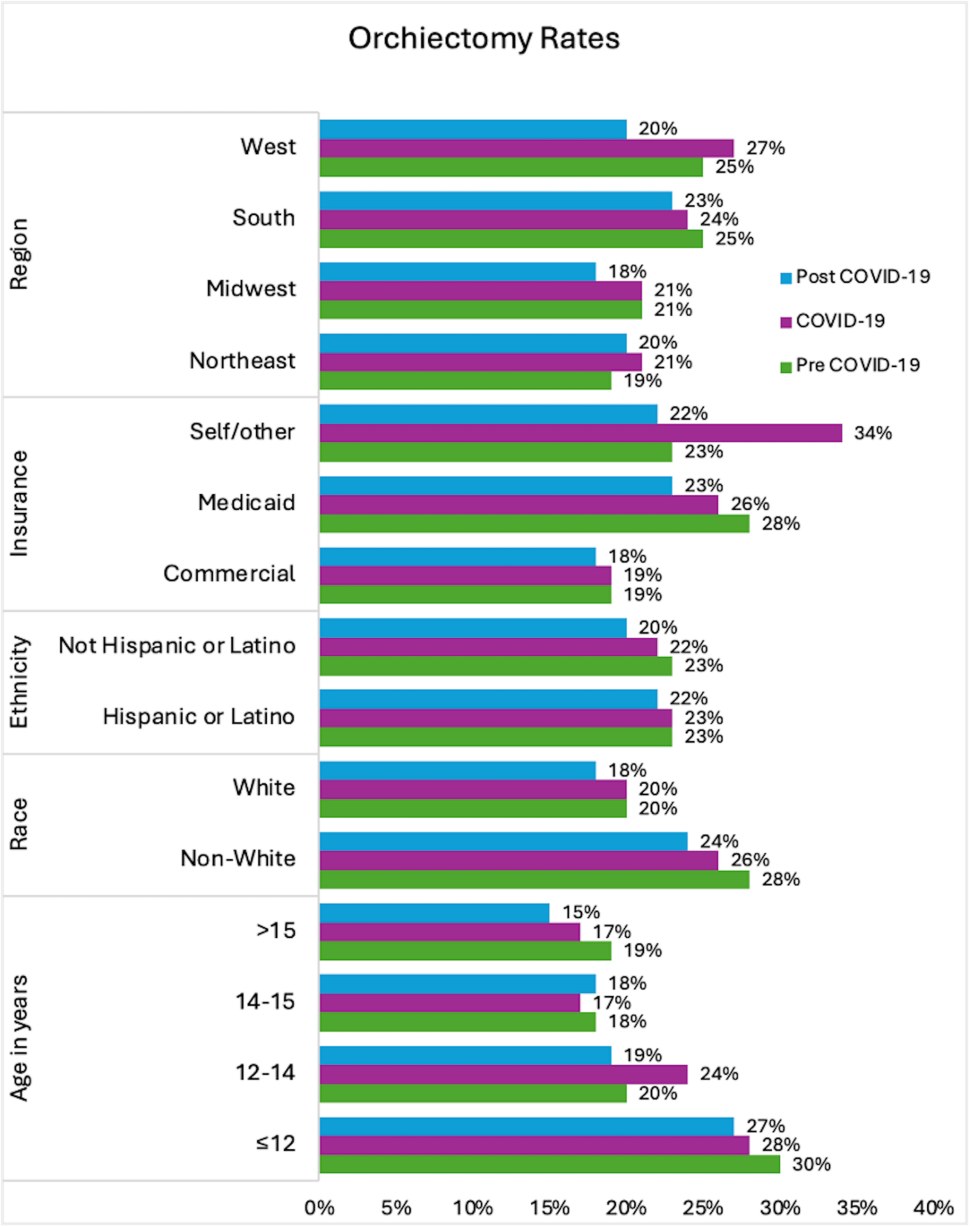
Orchiectomy rates by patient demographics.

Overall, rates of orchiectomy appear to decline over time (Figure 7), although there was no statistically significant difference in the odds of orchiectomy over time for any age group (Table 2). The overall orchiectomy rates showed a downward trend over time in all age groups, with an uptick in orchiectomy rates in younger patients (≤12 years of age) at the end of the post-COVID-19 period (Figure 8). Younger patients (≤12 years of age) consistently had the highest orchiectomy rates: 30% pre-COVID-19, 28% during COVID-19, and 27% post-COVID-19 (Figure 9). Younger patients had higher odds of orchiectomy compared to all other age groups both pre- and post-COVID-19. During COVID-19, younger patients had higher odds of orchiectomy, although this was statistically significant only when compared to patients aged 14-15 years and >15 years. Post-COVID-19, patients aged 12-14 years also had higher odds of orchiectomy compared to patients >15 years of age (Table 3). The lowest rates of orchiectomy were observed in patients aged 14-15 years (18% pre-COVID-19, 17% during COVID-19, and 18% post-COVID-19) and >15 years (15%, 17%, and 19%, respectively) (Figure 9).

Race

**Figure 10:**
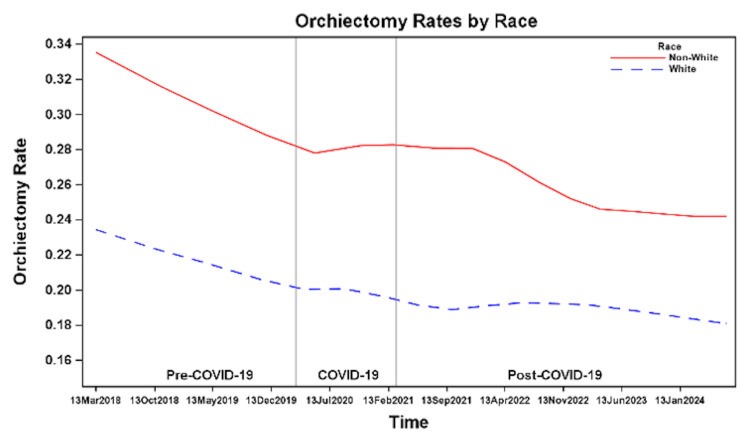
Orchiectomy rates before, during, and after the COVID-19 pandemic by race.

The overall rates of orchiectomy showed a downward trend over time in both White and non-White patients (Figure 10). However, there was no statistically significant difference in the odds of orchiectomy during COVID-19 or post-COVID-19 compared to pre-COVID-19 in either group (Table 2). Orchiectomy rates were consistently higher among non-White patients across all periods, 28% pre-COVID-19, 26% during COVID-19, and 24% post-COVID-19, compared to White patients with rates of 20%, 20%, and 18%, respectively (Figure 9). Non-White patients had significantly higher odds of undergoing orchiectomy across all time periods compared to White patients (Table 3).

Ethnicity

**Figure 11:**
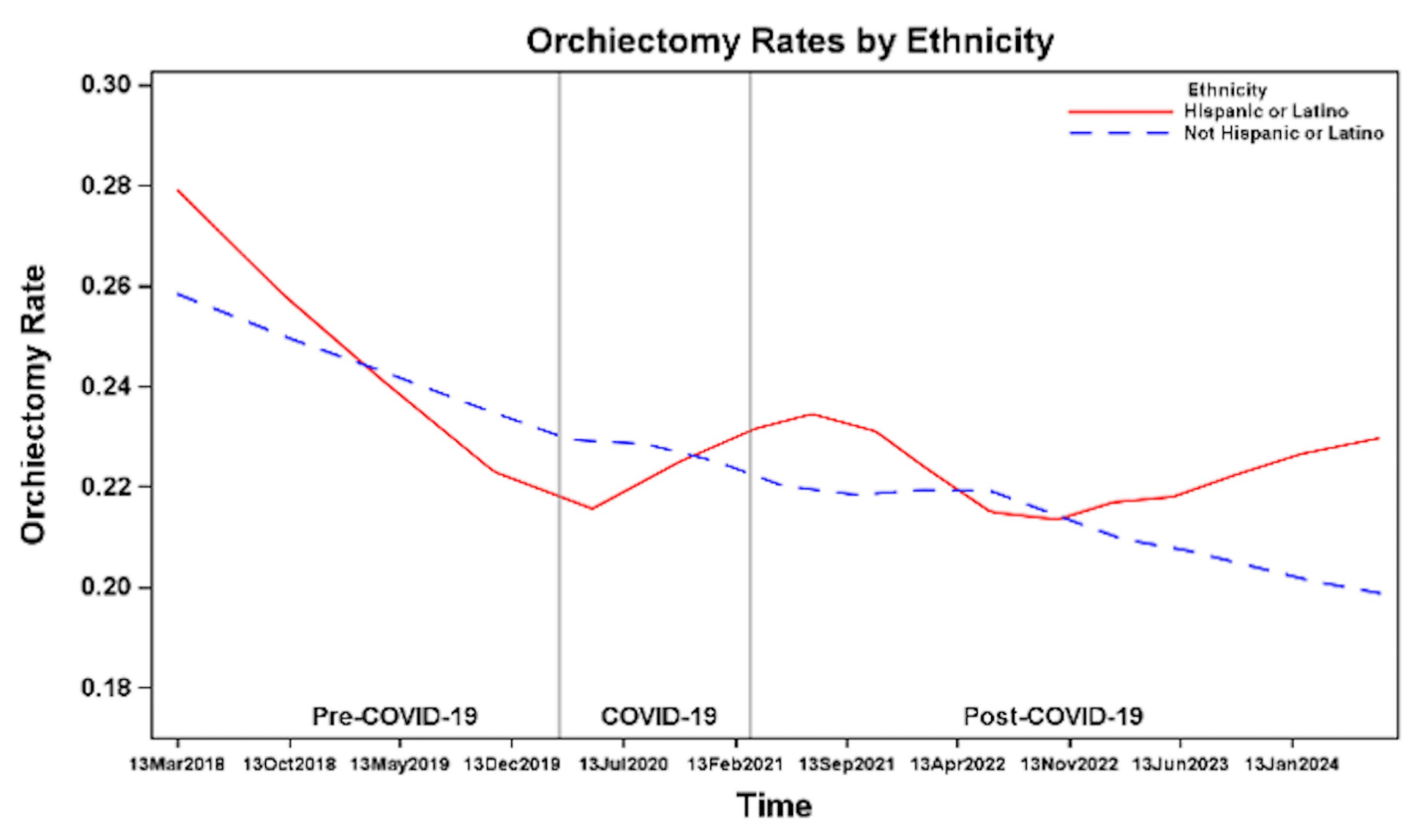
Orchiectomy rates before, during, and after the COVID-19 pandemic by ethnicity.

Non-Hispanic/Latino patients showed a steady downward trend in orchiectomy rates over time. In contrast, Hispanic/Latino patients exhibited a slight uptick in orchiectomy rates during COVID-19 and again at the end of the post-COVID-19 period (Figure 11). Among Hispanic/Latino patients, orchiectomy rates remained stable at 23% pre-COVID-19 and during COVID-19, and 22% post-COVID-19. In non-Hispanic/Latino patients, rates declined from 23% pre-COVID-19 to 22% during COVID-19 and 20% post-COVID-19 (Figure 9), and there was a statistically significant decrease in the odds of orchiectomy post-COVID-19 compared to pre-COVID-19 (OR 0.83, 95% CI 0.71-0.97, p=0.01) (Table 2). There was no statistically significant difference in the odds of orchiectomy between Hispanic/Latino patients and non-Hispanic/Latino patients across all time periods (Table 3).

Insurance

**Figure 12:**
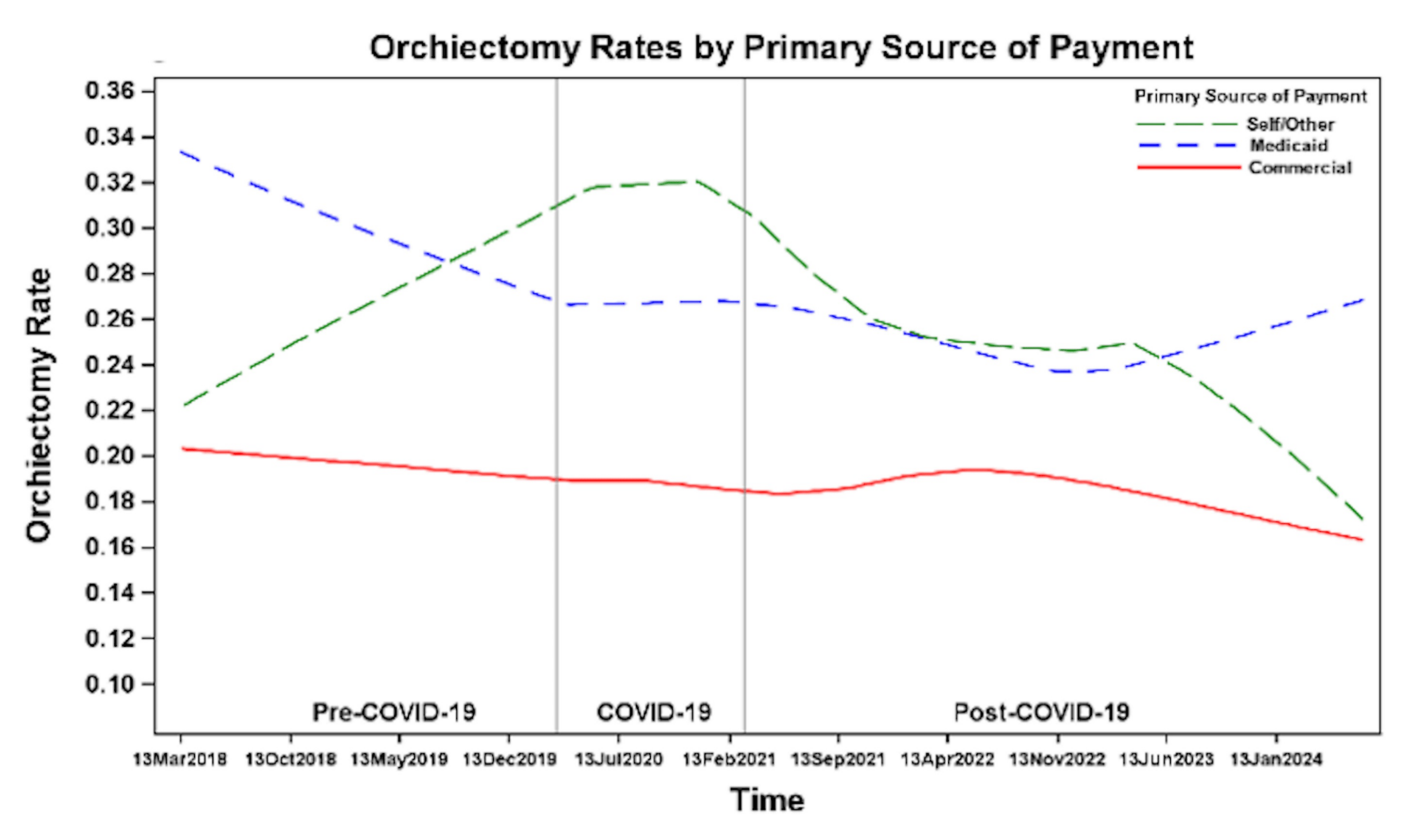
Orchiectomy rates before, during, and after the COVID-19 pandemic by insurance type.

Overall, orchiectomy rates remained stable for commercially insured patients and declined among patients with Medicaid, with lower odds of orchiectomy for patients with Medicaid post-COVID-19 compared to pre-COVID-19 (OR 0.8, 95% CI 0.67-0.95, p=0.006) (Table 2). Among patients who were self-insured or had other sources of insurance, there was an upward trend in orchiectomy rates beginning in the pre-COVID-19 period and peaking during the COVID-19 period, followed by a downward trend beginning in the mid-COVID-19 period and continuing into the post-COVID-19 period (Figure 12). Orchiectomy rates were lowest among patients with commercial insurance, at 19% pre-COVID-19 and during COVID-19, and 18% post-COVID-19 (Figure 9). Patients with commercial insurance had significantly lower odds of undergoing orchiectomy compared to those with Medicaid during all time periods (Table 3). Patients with commercial insurance also had significantly lower odds of undergoing orchiectomy compared to self-insured patients or patients with other sources of insurance during COVID-19 and post-COVID-19 (Table 3).

Region

**Figure 13:**
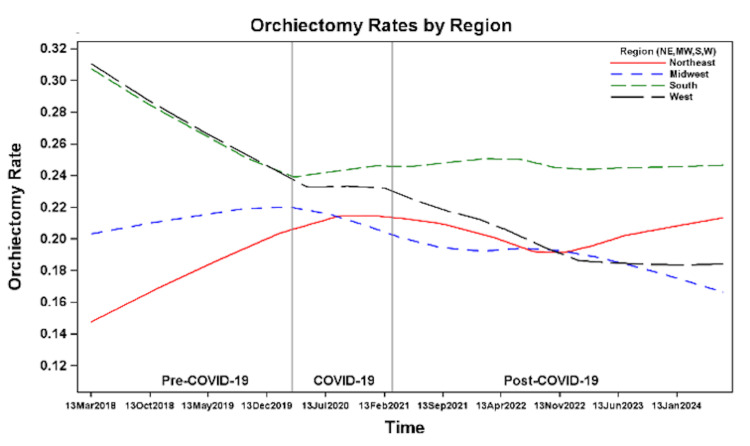
Orchiectomy rates before, during, and after the COVID-19 pandemic by geographic region.

Orchiectomy rates remained relatively stable in the Midwest and South, with an apparent upward trend over time in the Northeast and an apparent downward trend over time in the West (Figure 13). However, geographic region did not demonstrate a statistically significant association with orchiectomy rates between regions or across the pre-COVID-19, during COVID-19, and post-COVID-19 periods (Tables 2, 3).

**Table 2:** Odds of orchiectomy by patient factors. OR: odds ratio, CI: confidence interval.

Factor		Comparison	OR	95% CI	p-value
Age	≤12 years	COVID-19 vs pre-COVID-19	0.91	(0.67-1.26)	0.99
		Post-COVID-19 vs pre-COVID-19	0.86	(0.68-1.08)	0.42
	12-14 years	COVID-19 vs pre-COVID-19	1.22	(0.89-1.67)	0.46
		Post-COVID-19 vs pre-COVID-19	0.93	(0.73-1.19)	0.98
	14-15 years	COVID-19 vs pre-COVID-19	0.94	(0.54-1.62)	1
		Post-COVID-19 vs pre-COVID-19	0.98	(0.66-1.46)	1
	>15 years	COVID-19 vs pre-COVID-19	0.88	(0.53-1.44)	0.99
		Post-COVID-19 vs pre-COVID-19	0.77	(0.53-1.11)	0.32
Race	Non-white	COVID-19 vs pre-COVID-19	0.92	(0.68-1.25)	0.92
		Post-COVID-19 vs pre-COVID-19	0.82	(0.65-1)	0.092
	White	COVID-19 vs pre-COVID-19	1	(0.78-1.28)	1
		Post-COVID-19 vs pre-COVID-19	0.88	(0.73-1.1)	0.31
Ethnicity	Hispanic	COVID-19 vs pre-COVID-19	0.97	(0.66-1.42)	1
		Post-COVID-19 vs pre-COVID-19	0.93	(0.71-1.22)	0.93
	Non-Hispanic	COVID-19 vs pre-COVID-19	0.97	(0.79-1.18)	0.98
		Post-COVID-19 vs pre-COVID-19	0.83	(0.71-0.97)	0.01
Insurance	Commercial	COVID-19 vs pre-COVID-19	1.05	(0.82-1.35)	0.95
		Post-COVID-19 vs pre-COVID-19	0.94	(0.77-1.13)	0.8
	Medicaid	COVID-19 vs pre-COVID-19	0.91	(0.71-1.16)	0.69
		Post-COVID-19 vs pre-COVID-19	0.8	(0.67-0.95)	0.006
	Self/Other	COVID-19 vs pre-COVID-19	1.71	(0.92-3.18	0.107
		Post-COVID-19 vs pre-COVID-19	0.95	(0.59-1.53)	0.99
Region	Northeast	COVID-19 vs pre-COVID-19	1.08	(0.6-1.95)	1
		Post-COVID-19 vs pre-COVID-19	1.04	(0.66-1.64)	1
	Midwest	COVID-19 vs pre-COVID-19	1	(0.68-1.45)	1
		Post-COVID-19 vs pre-COVID-19	0.79	(0.59-1.06	0.21
	South	COVID-19 vs pre-COVID-19	0.95	(0.72-1.26)	1
		Post-COVID-19 vs pre-COVID-19	0.91	(0.75-1.13)	0.9
	West	COVID-19 vs pre-COVID-19	1.07	(0.7-1.64)	1
		Post-COVID-19 vs pre-COVID-19	0.75	(0.54-1.04)	0.13

**Table 3:** Factors associated with orchiectomy. OR: odds ratio, CI: confidence interval.

Factor	Time period	Comparison	OR	95% CI	p-value
Age	Pre-COVID-19	≤12 vs 12-14 years	1.69	(1.3-2.21)	< .001
		≤12 vs 14-15 years	1.96	(1.36-2.83)	< .001
		≤12 vs >15 years	1.83	(1.3-2.57)	< .001
		12-14 vs 14-15 years	1.15	(0.79-1.68)	0.76
		12-14 vs >15 years	1.08	(0.76-1.52)	0.95
		14-15 vs >15 years	0.93	(0.61- 1.43)	0.98
	COVID-19	≤12 vs 12-14 years	1.27	(0.92-1.76)	0.22
		≤12 vs 14-15 years	1.92	(1.2-3.07)	0.002
		≤12 vs >15 years	1.91	(1.23-2.97)	< .001
		12-14 vs >16 years	1.5	(0.95-2.39)	0.1
		12-14 vs 14-15 years	1.5	(0.98-2.31)	0.069
		14-15 vs >15 years	0.99	(0.58-1.73)	1
	Post-COVID-19	≤12 vs 12-14 years	1.57	(1.32-1.87)	< .001
		≤12 vs 14-15 years	1.72	(1.36-2.17)	< .001
		≤12 vs >15 years	2.04	(1.63-2.57)	< .001
		12-14 vs 14-15 years	1.1	(0.87-1.38)	0.75
		12-14 vs >15 years	1.3	(1.04-1.64)	0.016
		14-15 vs >15 years	1.19	(0.9-1.57)	0.37
Race	Pre-COVID-19	Non-White vs White	1.5	(1.19-1.93)	<0.001
	COVID-19	Non-White vs White	1.4	(1.04-1.88)	0.018
	Post-COVID-19	Non-White vs White	1.4	(1.2-1.64)	<0.001
Ethnicity	Pre-COVID-19	Hispanic vs Non-Hispanic	1.02	(0.79-1.33)	1
	COVID-19	Hispanic vs Non-Hispanic	1.03	(0.73-1.44)	1
	Post-COVID-19	Hispanic vs Non-Hispanic	1.15	(0.96-1.36)	0.17
Insurance	Pre-COVID-19	Commercial vs Medicaid	0.59	(0.48-0.74)	<0.001
		Commercial vs Self/Other	0.77	(0.5-1.17)	0.3
		Medicaid vs Self/Other	1.29	(0.85-1.96)	0.33
	COVID-19	Commercial vs Medicaid	0.69	(0.53-0.9)	0.003
		Commercial vs Self/Other	0.47	0.29-0.76)	<0.001
		Medicaid vs Self/Other	0.68	(0.42-1.11)	0.15
	Post-COVID-19	Commercial vs Medicaid	0.7	(0.61-0.81)	<0.001
		Commercial vs Self/Other	0.75	(0.58-0.98)	0.035
		Medicaid vs Self/Other	1.07	(0.83-1.4)	0.78
Region	Pre-COVID-19	Northeast vs Midwest	0.89	(0.45-1.75)	0.97
		Northeast vs South	0.73	(0.38-1.40)	0.6
		Northeast vs West	0.71	(0.34-1.46)	0.62
		Midwest vs South	0.82	(0.50-1.33)	0.72
		Midwest vs West	0.8	(0.45-1.42)	0.75
		South vs West	0.97	(0.56-1.68)	1
	COVID-19	Northeast vs Midwest	0.97	(0.47-1.98)	1
		Northeast vs South	0.83	(0.42-1.65)	0.89
		Northeast vs West	0.72	(0.34-1.54)	0.68
		Midwest vs South	0.86	(0.51-1.46)	0.88
		Midwest vs West	0.74	(0.40-1.39)	0.61
		South vs West	0.87	(0.48-1.56)	0.92
	Post-COVID-19	Northeast vs Midwest	1.17	(0.45-1.75)	0.9
		Northeast vs South	0.83	(0.38-1.40)	0.82
		Northeast vs West	0.98	(0.34-1.46)	1
		Midwest vs South	0.71	(0.50-1.33)	0.19
		Midwest vs West	0.84	(0.45-1.42)	0.83
		South vs West	1.19	(0.56-1.68)	0.81

## Discussion

This study examines national trends in the frequency of testicular torsion and orchiectomy rates before, during, and after the COVID-19 pandemic, as well as demographic and socioeconomic factors associated with orchiectomy, using the PHIS database. Our findings reveal notable trends in the number of testicular torsion cases per month and in orchiectomy rates across demographic, socioeconomic, and geographic groups over the study period. To our knowledge, this is the first study to characterize the demographics of patients presenting with testicular torsion before, during, and after the COVID-19 pandemic. Furthermore, it is the first to specifically assess factors associated with orchiectomy rates in the context of the COVID-19 pandemic.

The observed increase in the number of testicular torsion cases per month before and during the pandemic, followed by stabilization post-COVID-19, suggests a rise in case volume; however, the absence of population-level denominators precludes conclusions regarding true incidence. Shields et al. and Nelson et al. similarly described a rise in cases of testicular torsion at their institutions during COVID-19 [14,15]. However, the observed monthly increase in testicular torsion cases may reflect population growth rather than a true rise in incidence. Other published studies demonstrated no change in the incidence of testicular torsion during the pandemic compared to pre-pandemic [19].

In this nationally representative study, we observed variation in monthly testicular torsion cases by age, race, ethnicity, insurance status, and regional location. Cases were most frequent in patients aged 12-14, consistent with prior studies linking early adolescence to increased torsion risk, likely due to pubertal testicular growth and increased susceptibility [7,8]. Contrary to previous reports, White and non-Hispanic/Latino patients had a higher frequency of testicular torsion across all time periods than non-White and Hispanic/Latino patients. In contrast, Steward et al. found that Black/African American patients were three times more likely than Caucasian patients to experience testicular torsion [21]. Lee et al. similarly found that Black patients had the highest frequency of testicular torsion in their cohort [19]. Self-insured patients and patients with other sources of insurance had the lowest frequency of testicular torsion, which may reflect socioeconomic differences in access to insurance. The South had the highest frequency, potentially due to regional variations in population density and demographic composition.

We observed a lower orchiectomy rate of 22.5% compared with the 32%-42% reported in other nationally representative studies [2,7,8]. Studies assessing the impact of COVID-19 on orchiectomy rates have shown mixed results. Consistent with our findings, several reported no significant difference between pre- and during-COVID-19 periods [9,14,16,19]. In contrast, Pogorelić et al. and Frisenda et al. both found a statistically significant increase in orchiectomies during COVID-19 compared to pre-COVID-19 [18,22]. However, both studies were conducted in Europe, where COVID-19 precautions and clinical guidelines differed, which may partially account for the contrasting findings. A systematic review and meta-analysis by Pogorelić et al. found no significant difference in orchiectomy rates during COVID-19 compared to pre-COVID-19 [23].

Previous studies show orchiectomy rates vary by age, race, insurance, and region, with independent risk factors including younger age, Black race, and Medicaid insurance [7,8]. We identified similar demographic and socioeconomic factors associated with orchiectomy, which remained consistent during and after the COVID-19 pandemic. We found that patients ≤12 years consistently had the highest orchiectomy rates during all time periods, potentially due to delayed presentation, diagnostic challenges, or difficulty accessing care. Non-White patients and those with Medicaid, self-pay, or other insurance types had higher orchiectomy rates; these disparities may reflect differences in access to care or delays in presentation, although causality cannot be established with the available data.

Regional differences could also contribute to disparities in healthcare accessibility and management practices. Zhao et al. reported higher odds of orchiectomy in patients in the Midwest and South [7]. However, similar to our findings, Cost et al. did not find any geographic variation in orchiectomy rates [8]. Potential regional differences in orchiectomy rates are likely multifactorial and may include factors related to healthcare accessibility, management practices, and socioeconomic and cultural influences.

The overall downward trend in orchiectomy rates over time suggests earlier presentation and/or intervention in patients presenting with testicular torsion. However, persistent disparities in outcomes based on age, race, and insurance status underscore the need for targeted interventions to ensure timely access to care. Future research should focus on identifying specific barriers to testicular torsion diagnosis and treatment, as well as evaluating strategies to enhance early recognition, particularly in high-risk populations.

Our study has several limitations. As with all research using administrative databases, PHIS does not provide true incidence data, which limits the ability to make population-level inferences. To mitigate potential bias from the growing number of PHIS hospitals over time, we included only hospitals with complete data across the entire study period. Nevertheless, the patient population captured may not be fully representative of the broader U.S. pediatric population, particularly children from lower socioeconomic backgrounds, those without adequate insurance coverage, or those residing in rural areas. Demographic and socioeconomic characteristics of the PHIS population, as well as its coverage of the national pediatric population, should be considered when interpreting these findings.

Although we observed an increase in the number of testicular torsion cases per month, the lack of true incidence data precludes formal statistical comparisons. Case counts may be influenced by population growth, changes in diagnostic practices, shifts in treatment approaches, and regional differences in healthcare systems or referral patterns, which limits generalizability. Other important factors, such as time to presentation, urban versus rural hospital setting, and COVID-19-related healthcare system pressures (e.g., emergency department delays), could not be assessed with PHIS data.

While our analysis identified significant associations between race, insurance status, and the likelihood of orchiectomy, these findings must be interpreted with caution regarding causality. A primary limitation of using administrative data is the absence of granular clinical variables, most notably symptom duration and time to presentation. We are unable to account for confounding variables. Additionally, the retrospective, descriptive nature of this study precludes any definitive inferences regarding causation. Furthermore, while we identify significant temporal and demographic shifts in orchiectomy rates, these results should be interpreted as associations that warrant further investigation through prospective registries or population-based studies that include a true denominator for incidence calculation.

Finally, the “non-White” race category was not further subdivided due to small sample sizes of individual subgroups, which could obscure meaningful differences in outcomes. Despite these limitations, the study provides valuable insights into temporal trends and factors associated with testicular torsion and orchiectomy across a large, multi-center pediatric cohort.

## Conclusions

We observed an increased frequency of testicular torsion before and during the COVID-19 pandemic, which then plateaued. The overall orchiectomy rate slowly decreased over time and was not altered by the pandemic. During the study period, higher case volumes of testicular torsion were observed among patients aged 12-14 years, White patients, and non-Hispanic individuals; however, these findings represent differences in observed frequency rather than population-based risk. Younger age, non-White race, and non-commercial insurance might be associated with an increased risk of orchiectomy, but further studies are required to determine this association. Future directions include targeted public health education on symptoms of testicular torsion for vulnerable groups and identifying barriers to timely care.
